# Lattice Boltzmann simulation for phase separation with chemical reaction controlled by ultrasound field

**DOI:** 10.1371/journal.pone.0324607

**Published:** 2025-07-18

**Authors:** Heping Wang, Ying Lu

**Affiliations:** Department of Key Laboratory of Engineering Mathematics and Advanced Computing, School of Sciences, Nanchang Institute of Technology, Nanchang, Jiangxi, People’s Republic of China; Beni-Suef University, EGYPT

## Abstract

In this work, the phase separation behavior and pattern formation in binary fluids with chemical reactions controlled by ultrasonic radiation were systematically investigated. We incorporated the density-dependent Arrhenius equation into a novel and modified model for phase separation. The coupling effects of the pre-exponential factor K, density, and frequency on the phase separation under the condition of ultrasonic field-regulated chemical reactions were evaluated. 1) The rate of chemical reaction can be slowed down and even blocked by controlling the frequency of the ultrasonic field. 2)We have established a criterion for evaluating the competition between chemical reactions and the ultrasonic fields. When the value of pre-exponential factor K is greater than or equal to 10^−4^, phase separation is primarily regulated by the chemical reaction; otherwise, the ultrasonic field dominates the phase separation. 3) By analyzing the average structure factor, it was quantitatively proven that an increase in the frequency can significantly shorten the phase preservation period of the chemical reaction and ultrasonic radiation force and accelerate the merging of the separated phases into a larger phase. 4) We have successfully simulated the morphological evolution of phase separation regulated by traveling waves in the ultrasonic field.

## 1. Introduction

With the development of global industrialization, oily wastewater is becoming more and more harmful to the environment. Emulsified oil in oily wastewater is stably dispersed in water in the form of oil-water emulsion, which is extremely difficult to remove. Therefore, it is imperative to develop effective demulsification methods.

In recent years, many researchers have devoted themselves to applying ultrasonic radiation to phase separation. Zhang et al. [1] used a high-speed imaging technique to observe the phase separation process of water (H_2_O)-20% succinonitrile (SCN) immiscible solution within ultrasound field. Combining with numerical simulation, the effects of ultrasonic cavitation and acoustic streaming on the fragmentation and migration of secondary droplets were revealed. Luo et al. [2] reviewed current understanding and developments of phase separation technology based on ultrasonic standing waves (USWs). Otumudia et al. [3] investigated the effect of ultrasound on the removal of emulsion plugging in oil reservoirs using a glass micromodel. Ultrasound at low frequency (20 kHz) and power (100 watt) proved to be the most efficient condition to dislodge trapped emulsions in the micromodel pores, facilitate droplet coalescence and increase fluid recovery. The percentage of recovered emulsions increased to 58% when the micromodel was exposed to ultrasound (20 kHz, 100 watt), as opposed to 53.3% in the case without ultrasound. Song et al. [4] investigated the effects of initial temperature, ultrasonic amplitude, and time, as well as a diversity of additives on the ultrasonic dehydration rate of oily sludge demulsification. The optimal demulsification conditions are confirmed (temperature: 20 °C, ultrasonic amplitude: 100%, and ultrasonic time: 5 min), based on the effectiveness and economy of dehydration.

In the meantime, phase separation controlled by chemical fields has been effectively studied for accurately predicting the structural morphology. Li et al. [5] systematically study an immiscible binary system undergoing thermal/photo reversible reactions in theory. The dynamical evolution to trivial states witnesses a new type of sophisticated phase amplification phenomenon—temporary phase separation (TPS). Oya et al. [6] applied a density functional theory to the kinetics of phase separation affected by crosslinking reactions in density polymer solutions. Zwicker [7] focused on the tight interplay between phase separation and chemical reactions originating from thermodynamic constraints. Sun et al. [8] modified the traditional Brusselator model to incorporate the intermolecular interactions, based on which a systematic study is performed on the pattern formation mediated by chemical reaction and phase separation. It is found that if the chemical reaction dominates, the pattern formation will be inhibited by the phase separation while if the phase separation dominates, the chemical reaction will prevent, under certain conditions, the domain size from growing which results in dissipative patterns other than macroscopic phase separations.

Although the use of ultrasonic radiation force and chemical reaction for phase separation has respectively been fruitful, the phase separation in binary fluids with chemical reactions controlled by ultrasonic radiation has been scarcely explored. We have to underline that phase separation controlled simultaneously by chemical reactions and ultrasonic radiation occurs in everyday life. In addition, when an unbalanced phase separation and chemical reactions act simultaneously in fluid systems under ultrasonic field space, the phase separation becomes very complex and the respective numerical results differ from those referring to phase separation of mixed fluids without chemical reactions. Therefore, there is an urgent need to study the phase separation of such fluids. This study quantitatively investigates the effects of critical parameters in flow and ultrasonic fields (e.g., ultrasonic frequency and chemical reaction rate) on the phase separation, and reveals the morphological evolution of separated phases under varying conditions. The findings provide critical insights for demulsification and oily wastewater treatment.

Studies on phase separation typically employ either experimental methods or numerical simulations. Experimental approaches often face challenges in accurately regulating parameters and isolating external influences. Therefore, we consider the study of phase separation by numerical simulations. Although traditional computational fluid dynamics (CFD) method have been widely applied to simulate various multiphase flow problem [9], the microscale level presents unique challenges for computational fluid dynamics (CFD) techniques. The lattice Boltzmann method (LBM) has gained attention as a viable option owing to its straightforwardness and capability to manage intricate microstructures [10,11]. Its inherent parallelism enables efficient simulations using Graphics Processing Units (GPUs) to achieve high computational performance [12]. The mesoscale characteristics of the Lattice Boltzmann Method (LBM) enable it to capture many advantages similar to molecular dynamics, which enhances its suitability for modeling complex multiphase flow systems [13–15]. In addition, we specifically consider using concentration to control the rate of chemical reactions. However, it is well-known that controlling concentration through fluid dynamics is highly challenging. Therefore, we propose utilizing the propagation of acoustic fields to regulate concentration. The propagation of acoustic fields is a weakly compressible process, and LBM is precisely the approach suited for simulating weakly compressible fluids. Hence, the LBM has the potential to simulate a variety of problems in phase separation fluids of our work.

Along these lines, the main objective of this work was to find possible LBM modeling strategies in two important aspects: model validation and multiscale analysis for achieving better control. Our analysis can be used for microemulsion precipitation as a viable alternative for attaining phase separation control under chemical reaction and ultrasonic field conditions. Only under the application of a large enough frequency, the mixture can slowly undergo phase separation in the presence of an ultrasonic field and a chemical reaction. First, an example was provided and then, the large-scale spatial inhomogeneities produced by a periodic spinodal decomposition controlled by the competition between the chemical reaction and phase separation were discussed. By choosing a smaller pre-exponential factor, the inhomogeneity was eventually enhanced. On top of that, the different interfacial effects induced by chemical reactions between A and B in the presence of an ultrasonic field were examined and the importance of coupling the ultrasonic and concentration fields was demonstrated. The different morphologies of the phase separation were also explored by tuning the ultrasonic field frequency and the mixture concentration. The dynamic asymmetry of the ultrasonic field and the concentration field exhibited a strong influence on both the morphology and the coarsening of the separated phase. Various experimental works [16–18] have also shown that different mesoscopic structures of the separated phases can be formed when the mixture is subjected to phase separation and chemical reactions under ultrasonic fields. By varying the ultrasonic frequency and K-value, a density difference competition between the phase separation and the phase mixing induced by the concentration diffusion and chemical reaction rates, respectively, can occur. As a result, the resulting morphologies include interconnected structures (ISs), lamellar structures (LSs), and concentric phase-separated structures (CSs). At the same time, these behaviors cannot be described by performing simulations on time and space scales. Therefore, in addition to the qualitative description of the evolution of the phase separation, the different phases of the phase separation and the corresponding mechanisms were quantitatively described using Fourier phase transitions and structure factors.

**Table:** 

Nomenclature	
$P_{\alpha \beta }$	Pressure tensor
$\vec{u}$	Velocity
ρ	Total density
λ(ρ)	Bulk viscosity
$\eta_{mix}$	Mixture kinematic viscosity
Γθ	Macroscopic mobility
δμ	Chemical potential difference
$\eta_{A}$	Viscosity of component A
r	The rate of reaction
$f_{i}$	The first distribution function for the two-component system
$g_{i}$	The second distribution function for the two-component system
$\vec{e_{i}}$	Velocity vector in the i-th direction
$\vec{x}$	Location of the lattice point
$\tau_{f}$	Relaxation time
$f_{i}^{\left(eq\right)}$	The second equilibrium distribution function for the two-component system
$g_{i}^{\left(eq\right)}$	The second equilibrium distribution function for the two-component system
φ	Density difference
$p_{s}$	Acoustic pressure
$\rho_{s}^{A}\;\;(\;\;t\;\;)$	Acoustic density at the boundary
${\vec{u}}_{s}^{A}\;\;(\;\;t\;\;)$	Acoustic velocity at the boundary
$\omega_{s}$	wave number
$\rho_{s}^{\left(0\right)}$	ambient density
$\delta \rho_{s}$	Amplitudes of the density variations
$\delta {\vec{u}}_{s}$	Amplitudes of the velocity variations
$f_{i}^{Ta}$	Distribution function of the target flow
$\rho_{0}$	Undisturbed density of the fluid
σ	Distance measured from the beginning of the buffer zone
$\sigma_{m}$	A constant equal to 0.3
$D_{th}$	Thickness of the buffer
$c_{s}$	Speed of sound
$P_{e}$	Period
δx	Length scales
δt	Time scales

## 2 Methods

Phase separation behavior is modeled here through phase field equations, which can be associated with a free energy, corresponding pressure tensor, and chemical potential for the binary fluids. The dimensionless governing equations of the fluid flow [19] are:


$$\frac{\partial \rho }{\partial t}+\nabla \cdot (\rho \vec{u})=0$$ (1)

$$\frac{\partial (\rho \vec{u})}{\partial t}+\nabla \cdot (\rho \vec{u}\vec{u})=-\nabla P_{\alpha \beta }+\eta_{mix}\nabla^{2}(\rho \vec{u})+\nabla (\lambda (\rho )\nabla (\rho \vec{u}))$$ (2)

$$\frac{\partial \varphi }{\partial t}+(\vec{u}\cdot \nabla )\varphi =\Gamma \theta \nabla^{2}\delta \mu -(r_{1}+r_{2})(\varphi -\frac{r_{2}-r_{1}}{r_{1}+r_{2}})$$ (3)

where $P_{\alpha \beta }$, $\vec{u}$, ρ, λ(ρ), $\eta_{mix}$, Γθ, δμ are the pressure tensor, velocity, total density, bulk viscosity, mixture kinematic viscosity, the macroscopic mobility, the chemical potential difference, respectively. The mixed viscosity of binary systems can be determined by the following expression:


$$\eta_{mix}=\frac{\rho +\varphi }{2\rho }\eta_{A}+\frac{\rho -\varphi }{2\rho }\eta_{B}$$ (4)

where $\eta_{A}$ and $\eta_{B}$ denote the viscosity of components A and B, respectively. ρ is the sum of the initial densities of the two components, and $\rho =\rho_{A}+\rho_{B}$. φ is the initial density difference between the two components, and $\varphi =\rho_{A}-\rho_{B}$. After simplification, $\eta_{mix}=\frac{\rho_{A}}{\rho }\eta_{A}+\frac{\rho_{B}}{\rho }\eta_{B}$ can be derived. $\frac{\rho_{A}}{\rho }$ and $\frac{\rho_{B}}{\rho }$ are respectively the viscosity weights for component A and component B.

The reaction rate of components A and B with density change can be determined by the following equation [20]:


$$\begin{array}{c} r_{1}=Ke^{\left(x,\rho_{1}\right)}\\ r_{2}=Ke^{\left(x,\rho_{2}\right)} \end{array}$$ (5)

where the magnitude of the K value responds to the vigor of the chemical reaction.

### 2.1 Phase separation with reversible chemical reaction under constant temperature field

The free energy model was chosen as the lattice Boltzmann phase separation model [21–23].


$$\psi =\int \left(\phi (Te,\rho ,\varphi )+\frac{\kappa }{2}{\left(\nabla \rho \right)}^{2}+\frac{\kappa }{2}{\left(\nabla \varphi \right)}^{2}\right)$$ (6)

where the chemical potential difference and the bulk free-energy density at a temperature can be described as follows, respectively:


$$\delta \mu (\varphi ,\rho ,Te)=-\frac{\lambda }{2}\frac{\varphi }{\rho }+\frac{Te}{2}\ln(\frac{\rho +\varphi }{\rho -\varphi })-\kappa \nabla^{2}\varphi -\kappa Te(\nabla \varphi \nabla \frac{1}{Te})$$ (7)

$$\phi (Te,\rho ,\varphi )=\frac{\lambda }{4}\rho (1-\frac{\varphi^{2}}{\rho^{2}})-T\rho +\frac{Te}{2}(\rho +\varphi )\ln(\frac{\rho +\varphi }{2})+\frac{Te}{2}(\rho -\varphi )\ln(\frac{\rho -\varphi }{2})$$ (8)

The pressure tensions are shown below:


$$P_{\alpha \beta }=p_{0}\delta_{\alpha \beta }+\kappa (\frac{\partial \rho }{\partial x_{\alpha }}\frac{\partial \rho }{\partial x_{\beta }}+\frac{\partial \varphi }{\partial x_{\alpha }}\frac{\partial \varphi }{\partial x_{\beta }})$$ (9)

where $p_{0}=\rho Te-\frac{\kappa }{2}(\rho \nabla^{2}\rho +\varphi \nabla^{2}\varphi )-\frac{\kappa }{2}(|\nabla \rho {|}^{2}+|\nabla \varphi {|}^{2})$

**Fig 1:**
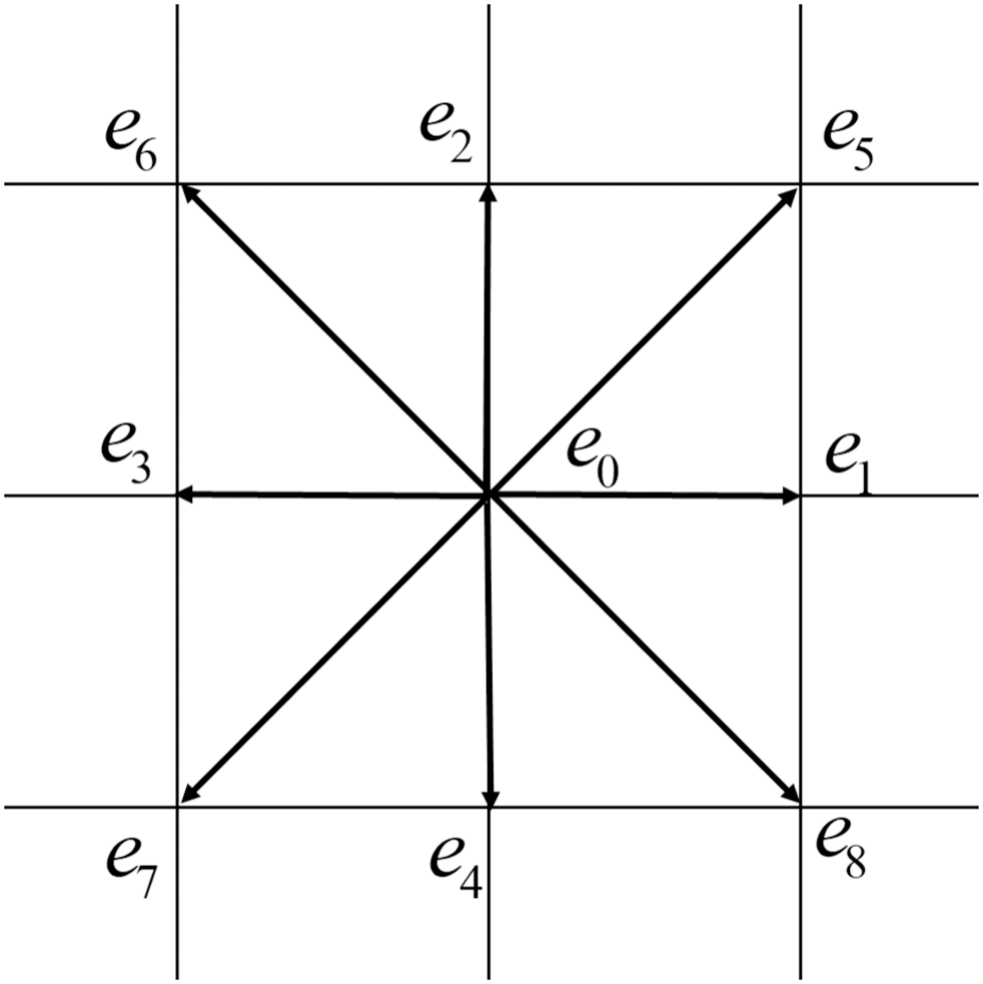
D2Q9 Lattice.


The distribution functions were associated with a lattice vector $e_{i}$ and model $e_{i}=\delta_{x}/\delta_{t}$ shown in Fig 1 [24]. This lattice framework forms the basis of our study, allowing precise research of the targeted phenomena [25].

$$\vec{e_{i}}=\left\{ \begin{array}{cc} @ll(0,0) & i=0,\\ c\left(\cos(i-1)\frac{\pi }{2},\sin(i-1)\frac{\pi }{2}\right) & i=1,2,3,4,\\ \sqrt{2}c\left(\cos(2i-1)\frac{\pi }{4},\sin(2i-1)\frac{\pi }{4}\right) & i=5,6,7,8 \end{array}\right.$$ (10)

### 2.2 Two-Dimensional LBGK model for concentration and temperature fields of phase separation

#### 2.2.1 Concentration field model.

The LBM with dual distribution function (DDF) is a numerical simulation method used for fluid dynamics [26]. In this study, the phase separation in a binary fluid was described by means of DDF. The evolution equations were discretized in space and time by using distribution functions $f_{i}$, and each distribution function was associated with a velocity vector $\vec{e_{i}}$:


$$f_{i}(\vec{x}+\vec{e_{i}}\delta t,\;\;\;\;t+\delta t)-f_{i}(\vec{x},\;\;\;\;t)=-\frac{1}{\tau_{f}}(f_{i}(\vec{x},\;\;\;\;t)-f_{i}^{\left(eq\right)}(\vec{x},\;\;\;\;t))$$ (11)

where $\vec{x}$ is the location of the lattice point, $\tau_{f}$ refers to the relaxation time, and $f_{i}^{\left(eq\right)}$ stands for the equilibrium distribution function.

Similarly, the second evolution equation for the two-component system can be obtained [27,28]:


$$g_{i}(\vec{x}+\vec{e_{i}}\delta t,\;\;\;\;t+\delta t)-g_{i}(\vec{x},\;\;\;\;t)=-\frac{1}{\tau_{g}}(g_{i}(\vec{x},\;\;\;\;t)-g_{i}^{\left(eq\right)}(\vec{x},\;\;\;\;t))$$ (12)

where the $\vec{x}$, $\tau_{g}$, $g_{i}^{\left(eq\right)}$ are similar to Eq.(11).

The macroscopic flow velocity $\vec{u}$, total density ρ, and density difference φ are provided below with respect to the two equilibrium distribution functions:


$$\rho =\sum_{i}f_{i}^{\left(eq\right)},$$ (13)

$$\rho u_{\alpha }=\sum_{i}f_{i}^{\left(eq\right)}e_{i\alpha },$$ (14)

$$\varphi =\sum_{i}g_{i}^{\left(eq\right)}.\;\;\;$$ (15)

The equilibrium density distribution function $f_{i}^{\left(eq\right)}$, as well as the higher order matrices $g_{i}^{\left(eq\right)}$, can be defined as shown below:


$$\sum_{i}f_{i}^{\left(eq\right)}e_{i\alpha }e_{i\beta }=P_{\alpha \beta }+\rho u_{\alpha }u_{\beta },\;\;\;\;$$ (16)

$$\sum_{i}g_{i}^{\left(eq\right)}e_{i\alpha }=\varphi u_{\alpha },\;\;\;\;\;\;$$ (17)

$$\sum_{i}g_{i}^{\left(eq\right)}e_{i\alpha }e_{i\beta }=\Gamma \delta \mu \delta_{\alpha \beta }+\varphi u_{\alpha }u_{\beta }.\;\;\;\;\;\;\;\;\;\;\;\;$$ (18)

This is because the macroscopic Eqs (1)–(3) must be satisfied to describe the dynamic moments of a binary liquid mixture. Here, the free energy lattice Boltzmann scheme was also applied to Eqs (16)–(18).

Through the Chapman-Enskog expansion, the continuity, Navier-Stokes (NS), and phase field Eqs (1)–(3) can be recovered via Eqs (11)–(12), and consequently, the following conclusions can be drawn:


$$\theta =\delta t(\tau_{g}-\frac{1}{2}),\;\eta_{mix}=\frac{\left(2\tau_{f}-1)\right)}{6}c^{2}\delta t,\lambda (\rho )=(\tau_{f}-\frac{1}{2})(\frac{2c^{2}}{3}-\frac{dp_{0}}{d\rho })\delta t$$ (19)

Where, θ represents the transport coefficient and $\tau_{g}$ is the relaxation time in the evolution equation (12). In weakly compressible fluids, the transport coefficient affects the speed and attenuation of sound wave propagation. $\eta_{mix}$ is the mixed viscosity of the two fluid components and $\tau_{f}$ is the relaxation time in the evolution equation (11). The mixed viscosity $\eta_{mix}$ increases with relaxation time $\tau_{f}$. The mixed viscosity determines the overall viscous dissipation characteristics of the multiphase fluid. λ(ρ) is the bulk viscosity in the governing equation (2), which describes the system’s response to density changes. These parameters collectively constitute the macroscopic mechanical response basis of multiphase flow systems.

#### 2.2.2 Acoustic model.

Because the pressure changes are small relative to the ambient pressure, sound waves can be introduced into the model [29,30]. The LBM applied here has the speed of cs=c/c3\nulldelimiterspace3. The acoustic pressure was found from the equation of the state: $p_{s}=(\;\;\rho -\rho_{0}\;\;)\;\;{c_{e}}^{2}$. The ultrasound field was introduced at the grid boundaries parallel to $\vec{e_{2}}$ This was done by setting the values of $\overset{―}{f_{i}}\;\;(\;\;\rho_{s}^{A}(\;\;t\;\;),\;\;{\vec{u}}_{s}^{A}\;(\;\;t\;\;)\;\;)$, where $\rho_{s}^{A}\;\;(\;\;t\;\;)$ and ${\vec{u}}_{s}^{A}\;\;(\;\;t\;\;)$ are the acoustic density and velocity at the boundary, respectively. A square grid was used with an integer number of wave-lengths in each direction. The values of the acoustic density and velocity can be therefore given by $\rho_{s}^{A}\;\;(\;\;t\;\;)=\rho_{s}^{\left(0\right)}$ and ${\vec{u}}_{s}^{A}\;\;(\;\;t\;\;)=\delta {\vec{u}}_{s}\;\;\sin\;\;(\;\;\omega_{s}t_{s}\;\;)$ for standing wave, respectively, and $\rho_{s}^{A}\;\;(\;\;t\;\;)=\rho_{s}^{\left(0\right)}+\delta \rho_{s}\;\;\sin\;\;(\;\;\omega_{s}t_{s}\;\;)$ and ${\vec{u}}_{s}^{A}\;\;(\;\;t\;\;)=\delta {\vec{u}}_{s}\;\;\sin\;\;(\;\;\omega_{s}\;\;t_{s}\;\;)$ for traveling wave, where $\omega_{s}$ is the wave number and $\rho_{s}^{\left(0\right)}$ states the ambient density; $\delta \rho_{s}$ and δu→s=(δρscs/δρscsρs(0)\nulldelimiterspaceρs(0),0) represent the amplitudes of the density and velocity variations, respectively.

### 2.3 Boundary conditions

In all simulations, the non-slip boundary condition was imposed at the top and bottom walls, as well as the inlet and outlet, which have been proposed for binary fluid systems [31]. Taking the upper boundary as an example, after the propagation the functions $f_{0}$, $f_{1}$, $f_{2}$, $f_{3}$, $f_{5}$, and $f_{6}$ are known on each site of the upper row, others can be defined as shown below:


$$\begin{array}{c} \rho =f_{0}+f_{1}+f_{3}+2(f_{2}+f_{6}+f_{5}),\\ f_{4}=\;\;f_{2},\\ f_{7}=\;\;f_{5}+\frac{1}{2}(f_{1}-f_{3}),\\ f_{8}=\;\;f_{6}-\frac{1}{2}(f_{1}-f_{3}), \end{array}$$ (20)

Here, the algorithm allowed to strict conserve the mass and momentum on the boundary walls.

#### 2.3.1 Acoustic boundary condition.

The top, bottom, and right boundaries of the fluid domain uses an absorbing boundary condition (ABC) [32], which is a transition buffer with a target flow prescribed at the outlet in the traveling wave field. The non-reflecting condition can be achieved by setting the distribution function of the target flow, $f_{i}^{Ta}$, to the equilibrium state, i.e., $\rho_{t}=\rho_{0}$ and $\vec{u_{t}}=0$, where $\rho_{0}$ is the undisturbed density of the fluid. For collision inside the transition buffer, an extra damping term was added to the collision equation of the single relaxation time BGK lattice Boltzmann scheme, as follows [33]:


$$f_{i}\;\;(\;\;x+\vec{e_{i}},\;\;t+\delta t\;\;)-f_{i}\;\;(\;\;x,\;\;t\;\;)=-\frac{1}{\tau_{f}}[f_{i}\;\;(\;\;x,\;\;t\;\;)-f_{i}^{\left(eq\right)}\;\;(\;\;x,\;\;t\;\;)\;\;]-\sigma \;\;(\;\;f_{i}^{\left(eq\right)}\;\;(\;\;x,\;\;t\;\;)-f_{i}^{Ta}\;\;(\;\;x,\;\;t\;\;)\;\;)$$ (21)

where σ=σm(δ/δD\nulldelimiterspaceDth)2 is the damping coefficient, $\sigma_{m}$ denotes a constant, normally equal to 0.3, σ refers to the distance measured from the beginning of the buffer zone, and $D_{th}$ signifies the thickness of the buffer. Inside the transition buffer, the amplitude of the outgoing waves is asymptotically attenuated and the reflections from the outside boundary are minimized. The thickness of the ABC buffer used in the model was 25 cells. The undisturbed dimensionless fluid density was set as $\rho_{0}=1.0$ for convenience. To ensure that the numerical stability and make the viscosity will be as small as possible, the calculated region was set to be less than two wave length. A high viscosity could result in accentuated wave dissipation, particularly at higher frequency components, and spurious directivity artifacts caused by the interaction between the viscous boundary layer and the rim of the calculated region. These arise from the definition of ρ, as described by Eq. (13)(13), which is not constant. In many fluid dynamic applications, as a disadvantage, compressibility errors are observed in LBM simulations [34]. This gives a speed of sound cs=(p0/p0ρ\nulldelimiterspaceρ)12. Additionally, for the binary-fluid model, the density variation will perturb the function gradient terms. Therefore, the phase separation induced by the density oscillation was thoroughly investigated in the following simulations propagating in the bound media.

#### 2.3.2 Meaning, precondition and assumptions of the acoustic model.

Here, the precondition of the LBM in the limit considering only low Mach numbers was used. There are a number of assumptions that restrict the applications of the LBM. More specifically, in the expression of the equilibrium distribution function, it was assumed that the Mach number M, $M=\frac{u}{c_{s}}$, is small. In the Taylor expansion of the Boltzmann equation the length and time scales of the simulations were also assumed to be small. Finally, the density variation must be also assumed to be small since the equations of motion are valid for an incompressible fluid. A further constraint originates from the fact that δx and δt should be small. Thus, the macroscopic scales of the wave are required to be much larger than the microscopic scales of the grid, for a wave of wavelength λ and period $P_{e}$: |δx|≪λ and $|\delta t|\ll P_{e}$. Altogether, with a suitable selection of λ and $P_{e}$ values, it is always possible to derive more stable simulation results.

In all simulations the propagation of acoustic signals was simulated through an immiscible binary fluid mixture. Acoustic pulses and waves were generated at the left boundary and reflected at the right boundary. The temperature Te in simulations was constant. The acoustic wave was fully reflected at the reflection boundary and absorbed at the top and bottom boundaries. The right boundary of vessel was the transducer, which was different from the vessel soaked in a cylindrical container filled with degassed water in the experimental apparatus. This made the process of phase separation be significantly shortened in our simulations.

## 3 Results and discussion

In this section, the pattern evolution of droplets under ultrasonic irradiation was demonstrated. This evolution is caused by two competing nonequilibrium phenomena in the presence of an ultrasonic field including chemical reactions and phase separation. In this case, the probability of collision between different components of the chemical reaction can be determined by the local equilibrium concentration, which is affected by the ultrasonic field. Therefore, the ultrasonic field is highly coupled with the chemical reaction. Dispersed droplets aggregate immediately after irradiation and a chevron pattern consisting of aggregates appears. Under given conditions, the frequency and K of the ultrasonic field contribute to the aggregation of droplets. Several features of the separated phase pattern can be summarized as follows: a) At lower frequencies, a square stripe pattern appears on the vessel wall and time migration leads to the formation of annular elongated droplets uniformly distributed. b) By increasing the frequency, annular elongated droplets gradually evolve into large round droplets that remain stable. c) K has a direct impact on emulsion separation. Particularly, a higher K induces the formation of square annular elongated droplets in solution, whereas a smaller K produces more dispersed small droplets. The acquired simulation results were compared and explored.

### 3.1 Model validation

**Fig 2:**
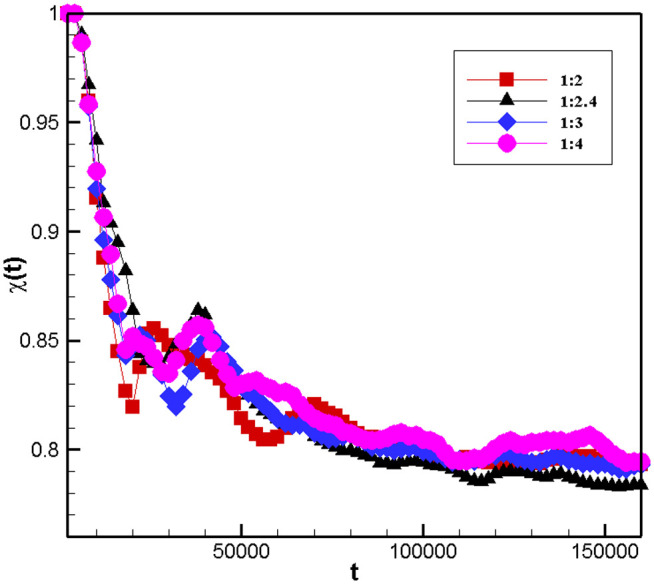
Spherically averaged structure factor S(k) versus wave number k for different grid numbers.

To examine the potential impact of grid on simulation results, we conducted a grid independence test. Grids with aspect ratios of 1:2, 1:2.4, 1:3, and 1:4 were utilized to examine the impact of grid number on phase separation under conditions of a density difference φ=0.5, a pre-exponential factor $K=10^{-4}$,and an acoustic field frequency f=0.1MHz\nolimits. Fig 2 indicates that the ultrasonic field can accelerate phase separation regardless of the grid number. Numerical simulation results in grids with different aspect ratios exhibit strong stability. Therefore, it can be concluded that the simulations conducted in this study demonstrate grid independence. To ensure the accuracy of the simulation results while improving computational efficiency, an aspect ratio of 1:2.4 was adopted for the grid.

In References [35] and [36], the free-energy model based on LBM has been demonstrated to accurately simulate the phase separation of emulsions and droplet coalescence behavior under the action of ultrasonic fields. To validate the reliability of our study, we conducted numerical tests and comparative analyses with the results reported by References [35] and [36].

In the phase separation process dominated by the ultrasonic field, the morphology of the separated phase tends to interconnected structures [35]; While in the chemically-driven phase separation process, the morphology of the separated phase primarily exhibits lamellar and ring-like structures [36]. It is worth noting that the simulation results of this study are in good agreement with morphological evolution laws of the phase separation controlled by two aforementioned physical field.

**Fig 3:**
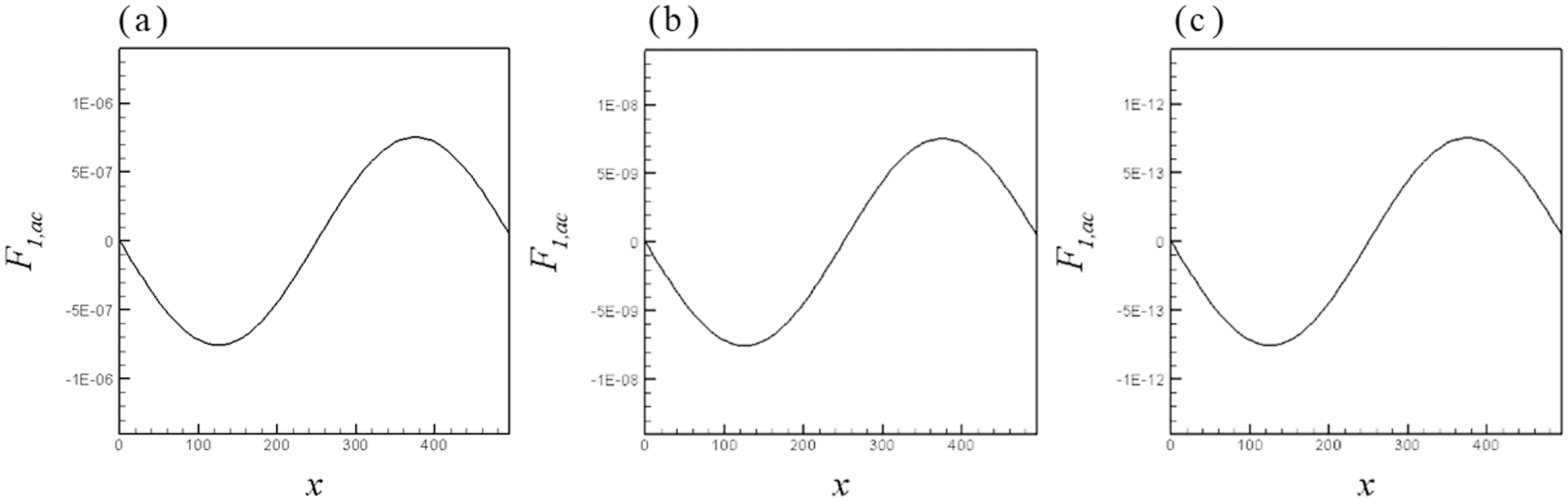
The spatial distribution of primary acoustic forces with $K=10^{-4}$ for (a) f=0.01MHz\nolimits, (b) f=0.1MHz\nolimits and (c) f=10MHz\nolimits.

Supplementary to the qualitative analysis, a quantitative study was carried out. Fig 3 illustrates the spatial distribution of primary acoustic forces acting on the separated phases under conditions of acoustic field frequency f=0.01MHz\nolimits, f=0.1MHz\nolimits and f=10MHz\nolimits. The results of our work are in good consistency with the research conclusions of Wang et al [35]. Specifically, it is manifested as follows: 1)Stable sine waves are generated at both the acoustic source and reflection ends. 2)The amplitude of the acoustic force decreases with the increase of frequency. 3)Although the amplitude of the primary acoustic force decreases under high-frequency conditions, due to the enhancement effect of frequency on the acoustic radiation force, the equivalent acoustic force received by the separated phase is more significant. This further confirms the rationality and credibility of the simulation.

### 3.2 Impact of the frequency on the separation of mixed emulsions with chemical reactions

**Fig 4:**
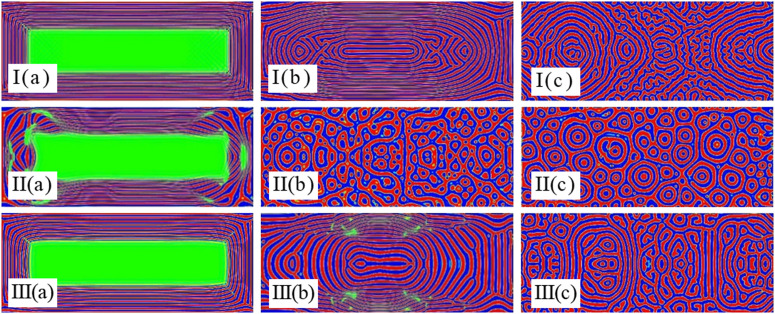
Coupling effect of the chemical reactions and ultrasonic fields on steady patterns with φ=0.5, $K=10^{-4}$ at times (a)t=1732×8, (b)t=1732×20 and (c)t=1732×100 for (I) f=0.01MHz\nolimits, (II) f=1MHz\nolimits and (III) f=10MHz\nolimits.

According to our previously published work [37], it is known that ultrasound frequency helps emulsions to undergo separation. Since the increase in the radiative and transverse radiative forces assists the droplets in aggregating in the standing wave field, the frequency of the acoustic field has an important impact on the separation and aggregation of emulsions [38–42]. Fig 4 depicts the pattern of chemically reactive solutions undergoing emulsion separation in a single wavelength, high frequency standing wave field. The sound intensity is varied by changing the frequency. The chemical reaction acts as an impedance in the emulsion to counteract the aggregation of the droplets in the standing wave field due to the increase in sound intensity. As can be seen in Fig 4: (I) the impact of the simulated chemical reaction impedance on the phase separation is greater than the impact of the acoustic field on the phase separation. (II) The impact of the simulated chemical reaction and ultrasonic field on the phase separation is comparable. (III) Simulated chemical reaction and ultrasonic field in which the ultrasonic field dominates. Based on the simulations presented in Fig 4, it can be argued that: 1) the phase separation is spreading from the wall to the center, and at first they are all regular rectangles. 2) when the chemical reaction dominates the interface, the final stable interface pattern is a twisted elongated strip. 3) when the chemical reaction is comparable to the ultrasonic field, the final stable interface is a circular shape. 4) when the ultrasonic field dominates the interface, it needs a more dense circular or even a droplet shape to keep the stabilization of the interface.

**Fig 5:**
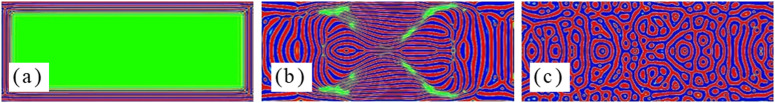
Coupling effect of the chemical reactions and ultrasonic fields on steady patterns with φ=0.65, $K=10^{-4}$, f=0.01MHz\nolimits at times (a)t=1732×5, (b)t=1732×15 and (c)t=1732×35.

As shown in Fig 5, holding other parameters constant, we added a calculation example with the initial density difference φ=0.65. Comparing with Fig 4I, it can be observed that: 1) The increase in density difference leads to a reduction in the frequency of collisions between components. This phenomenon significantly decreases the likelihood of transformation between components and shortens the phase separation time. 2) As the density difference increases, a large number of stripe structures perpendicular to the direction of the acoustic field are distributed at the sound source. The appearance of strip-shaped structures parallel to the direction of sound field in the acoustic wave oscillation region is significantly reduced. 3) Due to the increase in density difference, the intensity of chemical reactions between components weakens. The acoustic field plays a dominant role in the phase separation process, presenting more high-frequency patterns of phase separation.

### 3.3 Impact of K on the separation of mixed emulsions with chemical reactions

**Fig 6:**
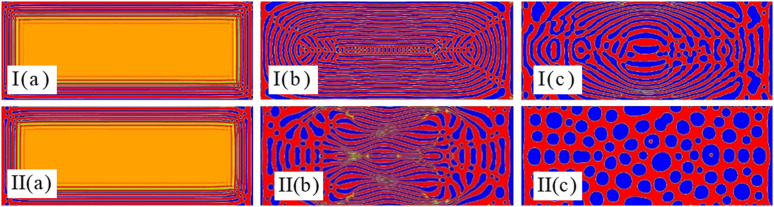
Coupling effect of the chemical reactions and ultrasonic fields on steady patterns with ρ=0.01, I(c) at times (a)t=1732×10, (b)t=1732×20 and $K=10^{-5}$ for (I) (a)t=1732×10 and (II) f=0.1MHz\nolimits.

**Fig 7:**
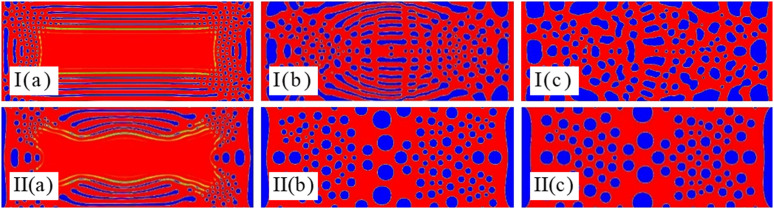
Coupling effect of the chemical reactions and ultrasonic fields on steady patterns with ρ=0.01, (c)t=1732×40 at times (a)t=1732×30, (b)t=1732×60 and II(a) for (I) II(c) and (II) f=0.1MHz\nolimits.

The magnitude of the K value responds to the vigor of the chemical reaction (i.e., the strength of the impedance in the presence of the ultrasonic field). As can be seen in Fig 4, the impedance generated by the chemical reaction has a relatively large effect on the transverse radiation force generated by the ultrasound field when the value of K is 10^−4^. As the value of K decreases, the ultrasonic field dominates and the morphology simulation is similar to that of the pure ultrasonic field. From Fig 6 it can be also observed that the impedance of the chemical reaction at a K value of 10^−5^ affects the transverse radiation force generated by the ultrasound field. In Fig 6I, it can be seen that even though the lower frequency morphology simulation is biased towards the low frequency morphology simulation of the ultrasonic field, the impedance generated by the chemical reaction is also present. This effect prevents the precipitation of droplets due to the ultrasonic field from aggregating to form spherical droplets, but instead stabilizes them to a long strip shape. As can be ascertained in Fig 7, the impedance of the chemical reaction at a K value of 10^−6^ has an almost negligible impact on the transverse radiative force generated by the ultrasound field. In Fig 7, the long droplets start first to precipitate from the surrounding wall, and then slowly converge into round droplets under the action of radiation force with the passage of time, whereas the small droplets merge with each other to form large droplets. This result is consistent with the morphology simulation of oil-water separation under the action of ultrasonic field [35].

**Fig 8:**
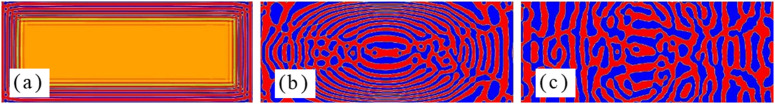
Coupling effect of the chemical reactions and ultrasonic fields on steady patterns with $K=10^{-6}$, (a)t=1732×30, f=0.01MHz\nolimits at times (c)t=1732×90, f=0.01MHz\nolimits and f=0.1MHz\nolimits.

Meanwhile, as shown in Fig 8, we also tested the case with an acoustic amplitude of $K=10^{-6}$. Comparing with Fig 6I, it can be concluded that (1) The increase in acoustic amplitude significantly shortens the separation time. (2) Due to the increase in acoustic field intensity, larger droplets are re-emulsified during the separation process. As a result, a large number of small droplets are dispersed among the larger droplets, presenting an irregular bicontinuous structure.

### 3.4 Impact of UHF on the separation of mixed emulsions by chemical reactions

**Fig 9:**
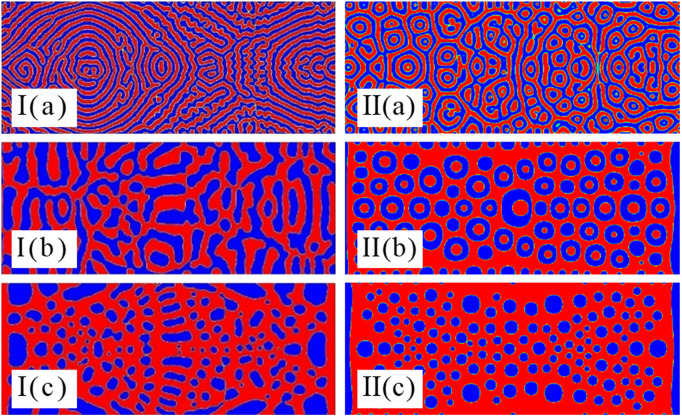
Coupling effect of the chemical reactions and ultrasonic fields on steady patterns with $K=10^{-5}$ for (a) $K=10^{-4}$, (b) (a)t=1732×10 and (c) $K=10^{-6}$ in process I with f=0.01MHz\nolimits and process II with f=20MHz\nolimits at time t=1732×80.

The solution morphology with chemical reaction under the action of UHF acoustic field can be simulated, which is difficult to realize in experimental research, and the following conclusions can be drawn by comparing Figs 9I and 9II: 1) When the K value is greater than 10^−4^, the chemical reaction dominates regardless of whether the sound field frequency is low or high or even ultra-high. (2) When the K value is 10^−5^, and the frequency is low frequency or high frequency, the chemical reaction and the radiation force generated by the acoustic field are comparable. Therefore, the interface droplet distribution is uniform. When the frequency is ultra-high frequency, the radiation force generated by the acoustic field is slightly larger than the impedance generated by the chemical reaction. Hence, the interface is distributed as uniform ring-shaped droplets. (3) When the value of K is less than 10^−6^, the impedance generated by the chemical reaction is negligible. As a result, the whole interface is controlled by the radiation force generated by the acoustic field, and the separated droplets are distributed along the propagation direction of the acoustic field.

### 3.5 Coupling effects of K and f on steady patterns

Regarding the impact of K and f on the morphological evolution, Tables 1 and 2 show the different states of phase separation under different $K=10^{-6}$ and f controls. Theoretically, the phase separation process can be roughly categorized into three distinct phases when a mixture undergoes a chemical reaction in the presence of an ultrasonic field: 1) A phase in which the chemical reactions dominate (i.e., the appearance of interconnecting structures (IS) when stabilized at the interface). 2) A stage in which the chemical reaction and the radiative force generated by the ultrasonic field hold. (i.e., concentric phase-separated structures (CPS) appear at the interface) 3) A stage in which the radiative force generated by the ultrasonic field dominates (i.e., droplet structures (DS) appear at the interface). As can be seen from Figs 4 and 7, the radiative force generated by the ultrasonic field helps to accelerate the chemical reaction to its maximum. Consequently, the phase separation reaches the phase-holding phase or the dominant phase more quickly as the frequency increases. From Tables 1 and 2, it can be seen that the radiation force generated by the ultrasonic field has a greater impact on the solution phase separation.

**Table 1:** The impact of thermal diffusion and viscosity on separated phase structures with φ=0.5.

K	f=0.01	f=0.1	f=1.0	f=10	f=20
$10^{-7}$	DS	DS	DS	DS	DS
$10^{-6}$	DS	DS	DS	DS	DS
$10^{-5}$	DS and CPS	DS and CPS	DS and CPS	DS and CPS	DS and CPS
$10^{-4}$	IS	IS	IS and CPS	IS and CPS	IS and CPS

**Table 2:** The impact of thermal diffusion and viscosity on separated phase structures with φ=0.6.

K	f=0.01	f=0.1	f=1.0	f=10	f=20
$10^{-7}$	DS	DS	DS	DS	DS
$10^{-6}$	DS	DS	DS	DS	DS
$10^{-5}$	DS and CPS	DS and CPS	DS and CPS	DS and CPS	IS and CPS
$10^{-4}$	IS	IS	DS and CPS	IS and CPS	IS and CPS

To further elucidate the coupling effect of the ultrasonic field and the chemical reaction on the phase separation, it was considered that the density difference may have an impact on the competition between the chemical reaction and the ultrasonic field, thus leading to a change in the pattern evolution. Therefore, control groups with different densities were simulated and the evolution is specifically shown in Table 2 Comparing Tables 1 and 2, it can be seen that density difference has no impact on the competition. Most importantly, the following conclusions can be drawn: the chemical reactions always dominate in the competition with the ultrasonic field when $10^{-6}$ > 10^−4^, the chemical reactions are in an even state in the competition with the ultrasonic field when K = 10^−5^, and chemical reactions are in a weak state in the competition with the ultrasonic field when K < 10^−6^. This means that when the rate of chemical reaction is much greater than the rate of the phase separation, the former is not localized throughout the process, but proceeds almost uniformly. The reason for this effect is that the chemical reaction continues to reduce the driving force for the phase separation, and there are no major fluctuations in the concentration. Meanwhile, when the rate of the phase separation is faster than the rate of the chemical reaction, the phase separation will first form the domain structure, while the chemical reaction occurs mainly at the domain interface, which may be due to the fact that the collision between the components occurring only at the interface.

### 3.6 Impact of the traveling wave on the separation of mixed emulsions by chemical reactions

**Fig 10:**
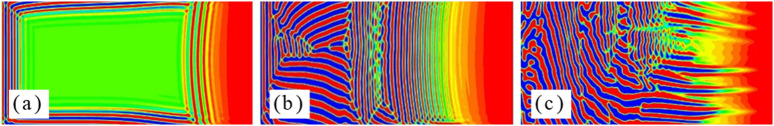
Coupling effect of the chemical reactions and the traveling wave on steady patterns with $K=10^{-4}$ at times (a)t=1732×4, φ=0.6 and K for f=0.01MHz\nolimits.

In order to compare with the impact of the standing wave field, we further explore the dynamic evolution process of emulsions under the regulation of the traveling wave field. Fig 10 shows the emulsion separation mode in the traveling wave field, and further demonstrates the influence of the competition between acoustic field radiative force and concentration. It can be seen from Fig 10 that: 1) Similar to the evolution process of the standing wave, in the initial stage, the sound source moves in the direction of the traveling wave to promote emulsion separation and form a strip structure. Secondly, with the extension of the sound field force, a band structure perpendicular to the direction of the sound field appears in the area where the sound field force is strong, while a band structure parallel to the direction of the sound field is preserved at the place far away from the sound source. Finally, with the evolution of time, the separated band structures collide with each other to form a double-connected structure. 2) The difference is that the evolution of the standing wave is continuous and there is no rebound superposition effect. This results in that the standing wave cannot prevent the chemical reaction of the emulsion like the traveling wave. Then, the emulsion continues to separate along the propagation direction of the sound field. 3) Since the rebound superposition effect cannot occur in the standing wave, the standing wave can make binary fluids with chemical reactions react more violently. In short, in the traveling wave field, the rate of chemical reaction is weak, which accelerates the emulsion separation.

### 3.7 Growth kinetics of phase separation

**Fig 11:**
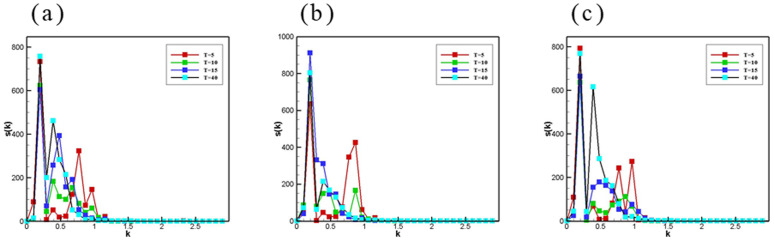
Spherically averaged structure factor S(k) versus wave number k for the procedures with $(a\mathrm{\backslash nolimits})\;K=10^{-4},\;\;\;\;f=0.1MHz$, (b)t=1732×12 and $(c\mathrm{\backslash nolimits})K=10^{-4},\;\;\;\;f=10MHz$. T is a multiple of $P_{e}$.

All curves shown in Fig 11 can be roughly divided into two distinct phases: the decomposition phase and the droplet growth phase. As displayed in Fig 11, the amplitude of the peak in the low-frequency case at wavelength K does not change in time, but the amplitude of the small-wavelength peak is larger than that of the large-wavelength peak. This behavior was indicative of the initial sharpening of the domain near the sound source and without a detectable phase separation taking place in the inner system. The origins of this effect are associated with the fact that the effects of the ultrasound field on the phase separation took place only near the sound source and reflection region. In the droplet growth phase depicted in Fig 11, the appearance of the first peak indicates the formation of a droplet-type phase structure in the vicinity of the acoustic source and reflection region. When a second peak appeared, this peak became the shoulder of the main peak and moved further to smaller wavelength values, indicating a subsequent growth of the droplet-type phase. The higher peak at the smaller wave vector corresponded to large domains close to the walls, while the other peak related to the smaller domain in the inner system. As time increased, the height of the first peak became bigger and the second peak tended to merge at larger wave numbers, suggesting a coarsening of the domains throughout the system. After that, the values of the larger wave vectors no longer seemed to drift to the left, but only oscillated in amplitude, suggesting a finite size effect.

Interestingly, the domain sharpening in the high-frequency condition was much faster than the low-frequency standing wave domain sharpening. This suggested that a highly frequent sound field accelerated a spinodal decomposition. In addition, the second peak appearing at the same wavelength was larger, indicating that the impact of high frequent sound field variation was more obvious farther from the sound source and reflection region. With time, more than two peaks appeared from the source region to the internal system and increased along the propagation direction of the ultrasonic field. As can be seen in Fig 11(c), the peaks in the high-frequency case were much larger than the peaks in the low-frequency case with similar corresponding wave numbers, which suggests that it is easier to obtain a higher degree of separation in the high-frequency case.

**Fig 12:**
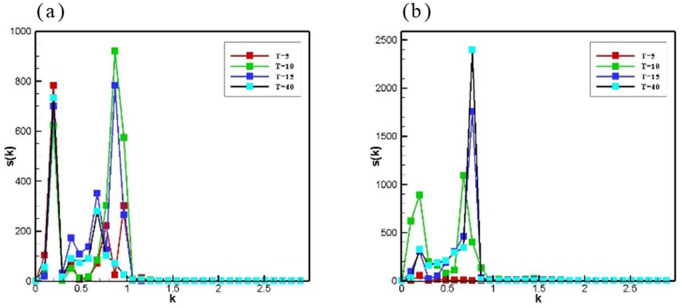
Spherically averaged structure factor S(k) versus wave number k for the procedures with $(a\mathrm{\backslash nolimits})\;K=10^{-4},\;\;\;\;f=0.1MHz$, $(b\mathrm{\backslash nolimits})\;K=10^{-4},\;\;\;\;f=1MHz$. T is a multiple of $P_{e}$.

The two plots of Fig 12 show that as the pre-exponential factor K decreased, the ultrasonic field radiative force gradually prevailed in the competition between the radiative force of the ultrasonic field and the antagonistic force generated by the chemical reaction. The ultrasonic field prevails in Fig 12(a), and with time the appearance of large droplets in the sound source out and reflection regions, two peaks in the plot emerge. The ultrasonic field and the chemical reaction lead to the production of a confrontation in Fig 12(b), so (b) is significantly slower than (a) in time evolution. This suggests that the force generated by the chemical reaction is opposing the radiative force generated by the ultrasonic field.

**Fig 13:**
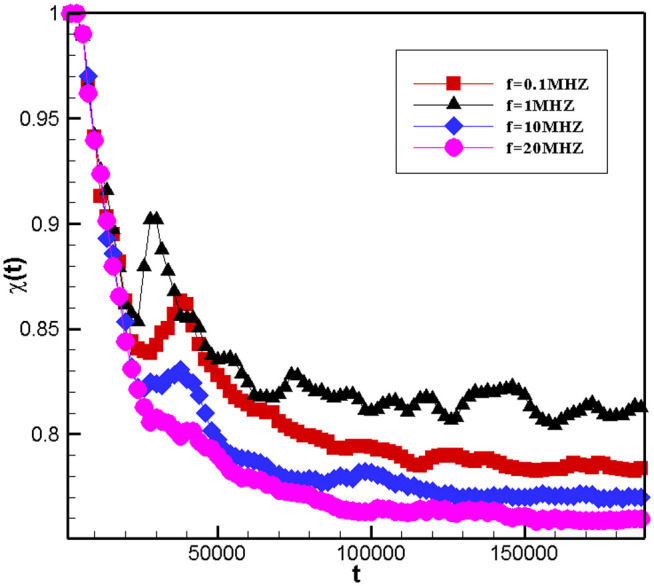
Time evolution of the degree of separation of emulsions with chemical reactions at different ultrasound frequencies.

Figs 12 and 13 represent time evolution plots of mixed emulsions of φ=0.5 at different ultrasonic frequencies and chemical reaction rates. The horizontal coordinate denotes the time and the vertical coordinate refers to the content ratio of the separated phase.

Fig 13 shows the variation of the emulsions with the same component ratio and carrying the same chemical reaction rate under different ultrasonic fields as a function of time. As can seen, 1) the ultrasonic field can increase the phase separation, which is increased with the increase in the frequency of the acoustic field. 2) The ultrasonic field can accelerate the speed of the phase separation. This also supports that the radiation force generated by the ultrasonic field will prevent the chemical reaction from occurring.

**Fig 14:**
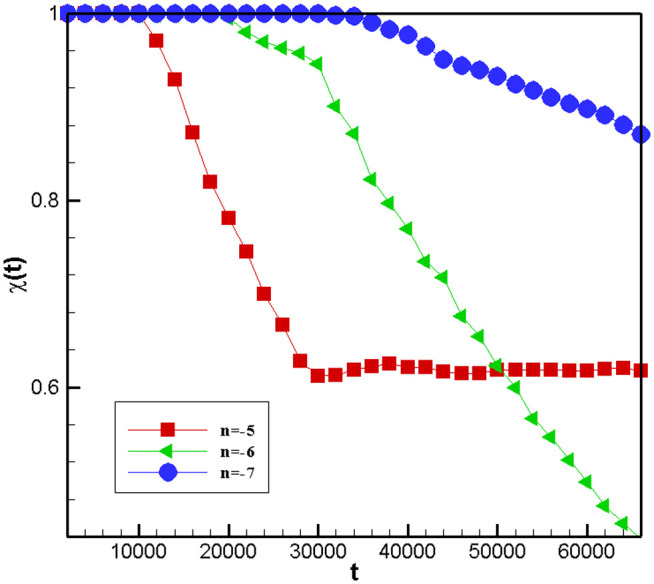
Time evolution of emulsion separations for different chemical reaction rates k at the same ultrasound frequency. n is the exponent of 10.

Fig 14 illustrates the time variation of emulsions with the same component ratio and different chemical reaction rates under the same ultrasonic field. From the acquired outcomes, it can be observed that: 1) the emulsion with different components and chemical reactions must have sufficient ultrasonic frequency to achieve phase separation. 2) As the rate of chemical reaction decreased, so does the rate of phase separation. However, the degree of separation increased.

## 4. Conclusions

In this work, it was demonstrated that LBM can be used to study phase separation patterns in ultrasonic fields. By increasing the frequency of the ultrasonic field, higher separation efficiencies can be realized. The morphology of solutions with chemical reactions was simulated based on ultrasonic fields in combination with the Arrhenius formula. From the simulated results, it was shown that many interesting patterns are generated by the coupling of the ultrasonic field and the chemical reaction. Several properties of the separated phase controlled by the concentration diffusion and chemical reactions, as well as the competition between ultrasonic fields, can be summarized as follows:

a) At $K>10^{-4}$, the chemical reaction always affects the diffusion of the concentration regardless of the frequency of the sound wave. The chemical reaction and the radiative force generated by the ultrasonic field are always in confrontation.
b) At $K<10^{-4}$, the time low frequency sound waves can fight and compete with the chemical reaction. However, as the frequency of the sound waves increases, the radiative force generated by the ultrasonic site prevails. Finally, the emulsion will produce separation.
c) The radiative force generated by the ultrasound field is in competition with the chemical reaction, and the ultrasound field can slow down the rate of chemical reaction. The rate of the chemical reaction can be controlled by controlling the frequency of ultrasound, and the ultra-high frequency can even block the chemical reaction. The standing wave can make the chemical reaction of the emulsion react more violently.
d) By analyzing the average structure factor, the phase separation process can be divided into a spinodal decomposition phase and a domain growth phase. From the quantitative analysis, it was demonstrated that an increase in the frequency significantly shortens the phase-holding phase of the chemical reaction and ultrasonic radiation force and accelerates the merging of the separated phases into larger ones.

In conclusion, it was shown that a phase separation can take place with a chemical reaction under an ultrasonic field, which satisfies the Arrhenius relation. Our work provides valuable insights for studying the evolution of separated phase structures and their control for more complex materials and outfields, such as ternary fluids and ultrasound fields.

## Supporting information

S1 Data The effects of the pre-exponential factor K, density difference, and ultrasonic frequency on phase separation behavior and pattern formation in binary fluid systems were evaluated in the dataset. The computational data were visualized to generate the figures presented in this work.
(ZIP)

S2 File This computational work was implemented in C++. The file contains all code required to generate the dataset (S1 Data).
(ZIP)
