# Supplementary material for: Lattice Boltzmann simulation for phase separation with chemical reaction controlled by ultrasound field
Source: PLoS One. 2025 Jul 18;20(7):e0324607. doi: 10.1371/journal.pone.0324607 (PMC12273979; doi:10.1371/journal.pone.0324607)
Supplement: S1 Data — The computational data were visualized to generate the figures presented in this work. (ZIP) [file pone.0324607.s001.zip › S1_Data/Supplementary Explanation.docx]

**Supplementary Explanation**

We implemented the computational framework in C++ and visualized the output data using “Tecplot”. The output data are all utilized for constructing the graphs in the manuscript. The detailed explanation is as follows.

“Data for Fig 2”: The data is used to investigate the variation of the spherically averaged structure factor with wave number for different grid numbers.

“Data for Fig 3”: The data shows the spatial distribution of primary acoustic forces for different ultrasonic field frequencies.

“Data for Fig 4”: The data is used to investigate the impact of the frequency on the separation of mixed emulsions with chemical reactions.

“Data for Fig 5”: The data is used to investigate coupling effect of the chemical reactions and ultrasonic fields on steady patterns with the initial density difference.

“Data for Fig 6”, “Data for Fig 7”: The data is used to investigate impact of K on the separation of mixed emulsions with chemical reactions.

“Data for Fig 8”: The data is used to investigate the coupling effect of the chemical reactions and ultrasonic fields on steady patterns with an acoustic amplitude of .

“Data for Fig 9”: The data is used to investigate impact of UHF on the separation of mixed emulsions by chemical reactions.

“Data for Fig 10”: The data is used to investigate impact of the traveling wave on the separation of mixed emulsions by chemical reactions.

“Data for Fig 11”: The data is used to investigate the variation of the spherically averaged structure factor with wave number for different ultrasonic field frequencies.

“Data for Fig 12”: The data is used to investigate the variation of the spherically averaged structure factor with wave number for different chemical reaction rates.

“Data for Fig 13”: The data shows time evolution of the degree of separation of emulsions with chemical reactions at different ultrasound frequencies.

“Data for Fig 14”: The data shows time evolution of emulsion separations for different chemical reaction rates at the same ultrasound frequency.
