# Supplementary material for: Lattice Boltzmann simulation for phase separation with chemical reaction controlled by ultrasound field
Source: PLoS One. 2025 Jul 18;20(7):e0324607. doi: 10.1371/journal.pone.0324607 (PMC12273979; doi:10.1371/journal.pone.0324607)
Supplement: S2 File — The file contains all code required to generate the dataset (S1 Data). (ZIP) [file pone.0324607.s002.zip › S2_File/Supplementary Explanation.docx]

**Supplementary Explanation**

We implemented the computational framework in C++. These codes are all utilized for constructing the graphs in the manuscript. The detailed explanation is as follows.

“Code for Fig 2”: The code is used to investigate the variation of the spherically averaged structure factor with wave number for different grid numbers.

“Code for Fig 3”: The code shows the spatial distribution of primary acoustic forces for different ultrasonic field frequencies.

“Code for Fig 4”: The code is used to investigate the impact of the frequency on the separation of mixed emulsions with chemical reactions.

“Code for Fig 5”: The code is used to investigate coupling effect of the chemical reactions and ultrasonic fields on steady patterns with the initial density difference.

“Code for Fig 6”, “Code for Fig 7”: The code is used to investigate impact of K on the separation of mixed emulsions with chemical reactions.

“Code for Fig 8”: The code is used to investigate the coupling effect of the chemical reactions and ultrasonic fields on steady patterns with an acoustic amplitude of .

“Code for Fig 9”: The code is used to investigate impact of UHF on the separation of mixed emulsions by chemical reactions.

“Code for Fig 10”: The code is used to investigate impact of the traveling wave on the separation of mixed emulsions by chemical reactions.

“Code for Fig 11”: The code is used to investigate the variation of the spherically averaged structure factor with wave number for different ultrasonic field frequencies.

“Code for Fig 12”: The code is used to investigate the variation of the spherically averaged structure factor with wave number for different chemical reaction rates.

“Code for Fig 13”: The code shows time evolution of the degree of separation of emulsions with chemical reactions at different ultrasound frequencies.

“Code for Fig 14”: The code shows time evolution of emulsion separations for different chemical reaction rates at the same ultrasound frequency.
